# TLR7-mediated inflammation drives PD-L1 upregulation and T cell exhaustion during influenza A virus infection

**DOI:** 10.1016/j.isci.2026.114776

**Published:** 2026-01-22

**Authors:** Mark A. Miles, Stella Liong, Felicia Liong, John J. O’Leary, Doug A. Brooks, Stavros Selemidis

**Affiliations:** 1 Centre for Respiratory Science and Health, School of Health and Biomedical Sciences, RMIT University, Bundoora, Melbourne, VIC 3083, Australia; 2 School of Pharmacy and Biomedical Sciences, College of Health, Adelaide University, North Terrace, Adelaide, South Australia 5001, Australia; 3 Discipline of Histopathology, School of Medicine, Trinity Translational Medicine Institute (TTMI), Trinity College Dublin, XW7X Dublin, Ireland; 4 Sir Patrick Dun’s Laboratory, Central Pathology Laboratory, St James’s Hospital, XW7X Dublin, Ireland; 5 CERVIVA Research Consortium, Trinity College Dublin, XW7X Dublin, Ireland

**Keywords:** biochemistry, cell biology, cancer

## Abstract

T cell dysfunction driven by dysregulated programmed cell death-1 (PD-1)/PD-ligand (PD-L) immune checkpoint signaling is associated with severe influenza A virus (IAV) infection. While this pathway limits immunopathology, it can suppress antiviral immunity and promote T cell exhaustion. We investigated the role of toll-like receptor 7 (TLR7), a viral RNA sensor, in regulating PD-1/PD-L-mediated T cell responses during IAV infection. Using wild-type and TLR7-deficient mice, we show that TLR7 activation enhances early antiviral T cell responses but subsequently increases PD-L1/PD-L2 expression, promoting T cell exhaustion at later stages of infection. This was associated with higher lung viral loads and increased expression of exhaustion-related genes. Mechanistically, TLR7 regulated PD-L1 expression indirectly via cytokine signaling, rather than directly affecting PD-1 expression. These findings identify TLR7 as a key upstream modulator of immune checkpoint signaling during IAV infection and suggest that targeting TLR7, alone or with checkpoint inhibitors, may boost antiviral immunity and reduce T cell exhaustion.

## Highlights

• During IAV infection, TLR7 drives cytokine-dependent PD-L1 and PD-L2 expression
• TLR7 boosts early T cell activation but promotes later checkpoint-mediated dysfunction
• TLR7-induced PD-L expression is inflammatory-driven, not T cell-intrinsic
• TLR7 deficiency limits checkpoint signaling and preserves antiviral T cell function

## Graphical abstract

**Figure:**
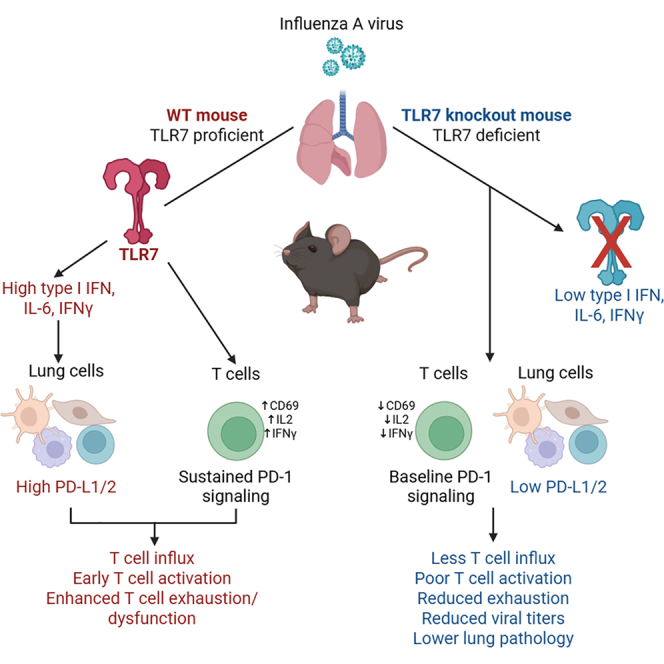

## Introduction

Influenza A virus (IAV) remains a leading cause of severe respiratory illness and global mortality. Beyond acute pathology, IAV can induce prolonged immune dysregulation, particularly within the lung, where persistent inflammation may impair tissue repair, promote secondary disease, and alter immune surveillance. The balance between effective antiviral immunity and immune-mediated damage is tightly governed by regulatory pathways that modulate T cell activation and exhaustion. Globally, unresolved IAV infections contribute to an estimated 294,000–581,000 deaths, annually.^1^ Effector T cells are important for targeting IAV-infected cells for destruction and secreting inflammatory cytokines and chemokines to enhance immune activation. These T cell mechanisms are crucial for viral clearance and the development of long-lasting immune memory.^2^ However, because effector T cells are inherently cytotoxic, their activation must be tightly regulated to prevent immunopathology, tissue damage, and chronic dysfunction.^3^ If left uncontrolled, extensive immune responses during respiratory infections can worsen disease pathology and predispose to long-term poor respiratory health, and other chronic inflammatory conditions such as autoimmunity and cancer.^4^^,^^5^

A major pathway controlling T cell activity is the programmed cell death-1 (PD-1)/PD-ligand (PD-L) immune checkpoint axis. PD-1 is transiently upregulated during T cell activation and binds to its ligands PD-L1 or PD-L2 to suppress proliferation, cytokine production, and cytotoxicity.^6^^,^^7^ This pathway maintains immune homeostasis,^8^^,^^9^ but in the setting of chronic antigen exposure or persistent inflammation, such as in cancer, PD-1 expression becomes sustained, leading to T cell exhaustion and functional decline.^10^ These exhausted T cells are marked by elevated levels of co-inhibitory receptors including PD-1, cytotoxic T-lymphocyte antigen 4 (CTLA-4), and T cell immunoreceptor with immunoglobulin and immunoreceptor tyrosine-based inhibitory motif domains (TIGIT), and exhibit a unique transcriptomic signature distinct from functional effector or memory T cells.^11^ Encouragingly, therapeutic blockade of PD-1 signaling in cancer can reinvigorate exhausted T cells and improve antitumor responses and survival.^12^

The targeting of PD-1 pathways during IAV infection to reduce acute and long-term impacts of the disease is an ongoing area of research. During the acute phase of IAV infection, T cells upregulate PD-1, which can diminish their effector activity.^13^^,^^14^^,^^15^ While this may impair viral clearance, it can be protective against excessive immunopathology, particularly in neonates.^16^ Regardless, PD-1 blockade during IAV infection or vaccination improves T cell function, viral clearance, and memory formation.^13^^,^^15^^,^^17^ However, complete loss of PD-1 such as in knockout (KO) mice can impair IAV-specific memory CD8^+^ T cells^18^ due to early T cell hyperactivation and subsequent death. This highlights the significance of ligand engagement for appropriate PD-1 signaling. Importantly, IAV infection in airway epithelial cells upregulates surface PD-L1 expression to help suppress T cell immunity.^15^^,^^19^ Together, these findings suggest that IAV infection enhances the expression of both PD-1 on T cells and PD-1 ligands on surrounding cells, highlighting the potential for enhanced PD-1/PD-L interactions to evade host immunity during infection.^20^

T cell activation during IAV infection relies on upstream innate immune signals to effectively clear the virus and establish immunological memory. Toll-like receptor 7 (TLR7) primarily senses IAV single-stranded RNA (ssRNA) leading to the initiation of proinflammatory and antiviral responses. Given its abundant expression in macrophages, plasmacytoid dendritic cells, and B cells, TLR7 supports the rapid development of adaptive immunity by facilitating antigen presentation and regulating cytokine production to promote T cell differentiation, effector activity, and immunological memory.^21^ Furthermore, T cells also express TLR7, although not as high as the aforementioned cells, and its direct activation in T cells can improve proliferation and effector activity.^22^^,^^23^^,^^24^ Importantly, therapeutic activation of TLR7 has been used to boost host immunity and improve the efficacy of PD-1/PD-L checkpoint blockade therapy in cancer.^25^^,^^26^^,^^27^^,^^28^ TLR7 agonists can also be used as vaccine adjuvants to improve effector T cell activation by modulating PD-1 expression.^29^ However, TLR7 hyperactivation can provoke pathogenic “cytokine storms,”^30^^,^^31^^,^^32^^,^^33^^,^^34^ and exacerbated inflammation during IAV infections is associated with increased PD-1 expression on IAV-specific CD8^+^ T cells leading to worsened outcomes.^14^ This raises the possibility that TLR7-mediated hyperinflammatory responses during IAV infection could alter immune checkpoints and impair T cell function. Indeed, direct stimulation of TLR3 (which detects double-stranded RNA viral intermediates), TLR7 or TLR9 (which detects unmethylated CpG DNA), has been shown to upregulate PD-L1 on endothelial, epithelial, and dendritic cells in a cell-intrinsic manner,^35^^,^^36^^,^^37^ implying that TLR signaling may impact T cell functionality via PD-1/PD-L regulation.

This study aimed to define the role of TLR7 in regulating the PD-1/PD-L axis during sublethal IAV infection. We found relative differences in the PD-1 and PD-L expression in the lungs of wild-type (WT) and TLR7 KO mice across infection, revealing a dominant T cell exhaustion signature driven by TLR7. TLR7 deficiency directly suppressed PD-L1 expression, but not PD-1, due to attenuated cytokine responses. These findings define a TLR7-dependent mechanism of PD-L1 regulation during IAV infection and suggest that innate immune sensing of viral RNA can shape checkpoint dynamics and promote T cell dysfunction.

## Results

### TLR7 deficiency limits T cell regulatory gene expression during IAV infection

**Figure 1:**
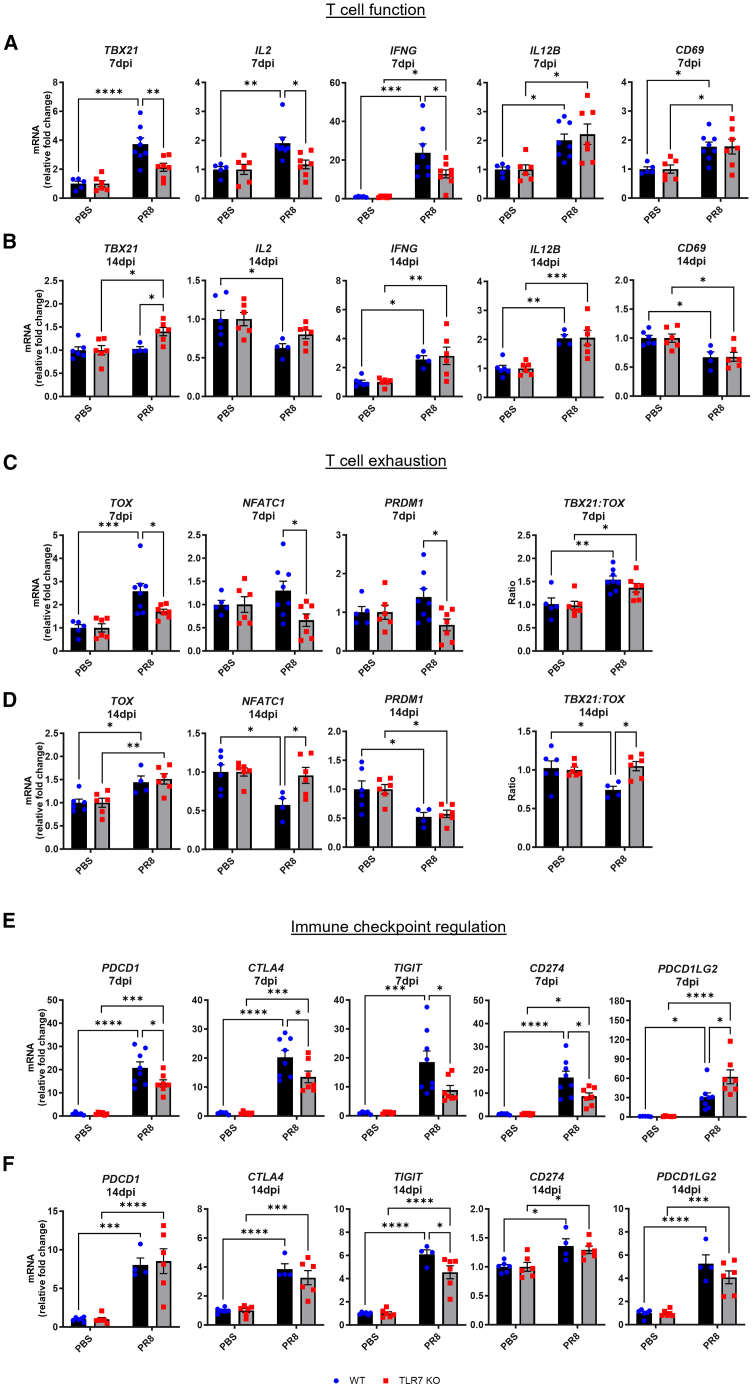
Altered gene expression of T cell regulation markers in the lungs of TLR7-KO mice following IAV infection WT C57BL/6 or TLR7-KO mice were intranasally infected with PR8 (50 PFUs) or mock infected with PBS. Lungs were harvested after 7 or 14 days and gene expression analysis was performed. mRNA expression of T cell (A and B) function, (C and D) exhaustion or (E and F) suppression markers were measured and expressed relative to RPS18 housekeeping as a fold-change above uninfected controls of each mouse genotype. Data are expressed as mean ± SEM (7 dpi: WT PBS *n* = 5, WT PR8 *n* = 8, TLR7-KO PBS *n* = 6, TLR7-KO PR8 *n* = 7; 14 dpi: WT PBS *n* = 5, WT PR8 *n* = 4, TLR7-KO PBS *n* = 5, TLR7-KO PR8 *n* = 6). Statistical analysis was conducted using two-way ANOVA test followed by Tukey’s post hoc test for multiple comparison test (∗*p* < 0.05, ∗∗*p* < 0.01, ∗∗∗*p* < 0.001, ∗∗∗∗*p* < 0.0001).

To better understand how TLR7 influences T cell functionality during IAV infection, we analyzed the expression of genes associated with T cell function, exhaustion, and immune checkpoint regulation in mouse lung tissue at 7 and 14 days post infection (dpi). These timepoints represent the early and established phases of the T cell response to PR8 infection, during which PD-1 expression typically increases on virus-specific T cells.^13^ A sublethal dose of the PR8 IAV strain was used to allow all mice to recover from the acute effects of infection. Both WT and TLR7-KO mice exhibited transient body weight loss following infection and returned to baseline by 14 dpi, with TLR7-KO mice showing a slightly delayed onset and recovery compared to WT mice as previously shown.^31^ Consistent with prior observations, TLR7-KO mice showed impaired TLR7 responses, reduced airway immune cell infiltration, decreased lung histopathology, and lower lung viral titers compared to WT controls (Figure S1). At 7 dpi, WT mice exhibited upregulation of canonical T cell activation genes including *TBX21*, *IL2*, *IFNG*, *IL12B*, and *CD69* (Figure 1A). In contrast, TLR7-KO mice showed significantly lower expression of *TBX21*, *IL2*, and *IFNG*, while *IL12B* and *CD69* levels were comparable to WT. By 14 dpi, both genotypes showed elevated *IFNG* and *IL12B*, and reduced *IL2* and *CD69*. Notably, *TBX21* expression was higher only in infected TLR7-KO mice at this later time point (Figure 1B), suggesting compensatory regulation in the absence of TLR7 signaling.

We then assessed markers of T cell exhaustion. *TOX*, a key exhaustion regulator,^38^ was significantly upregulated at 7 dpi in WT but not in TLR7-KO lungs (Figure 1C). Transcription factors *NFATC1* and *PRDM1*, which influence T cell exhaustion and activation,^39^^,^^40^^,^^41^ were also reduced in TLR7-KO mice. By 14 dpi, both genotypes showed similar *TOX* upregulation and *PRDM1* downregulation. However, *NFATC1* was significantly reduced only in WT mice (Figure 1D). To evaluate the balance between effector activation and exhaustion, we calculated the *TBX21:TOX* ratio. At 7 dpi, both genotypes showed a strong bias toward activation. However, by 14 dpi, this ratio declined markedly in WT mice, consistent with a shift toward exhaustion, while TLR7-KO mice retained a more activation-skewed profile. Checkpoint gene expression also differed between genotypes. At 7 dpi, infection increased lung expression of *PDCD1 (PD-1)*, *CTLA-4*, *TIGIT*, and their ligands *CD274* (encoding PD-L1) and *PDCD1LG2* (encoding PD-L2) in both groups (Figure 1E). However, TLR7-KO mice had significantly lower expression of all except *PDCD1LG2*, which was higher. At 14 dpi, expression of these checkpoint genes remained elevated but decreased from earlier levels; *TIGIT* remained significantly lower in TLR7-KO mice (Figure 1F).

Overall, TLR7 deficiency reduces the expression of genes associated with T cell activation, exhaustion, and checkpoint regulation during early IAV infection. By late infection, some differences persist, but only WT mice exhibit a transcriptional profile indicative of T cell exhaustion.

### Reduced PD-1+ T cell numbers in TLR7 KO lungs reflect impaired recruitment, not altered expression

**Figure 2:**
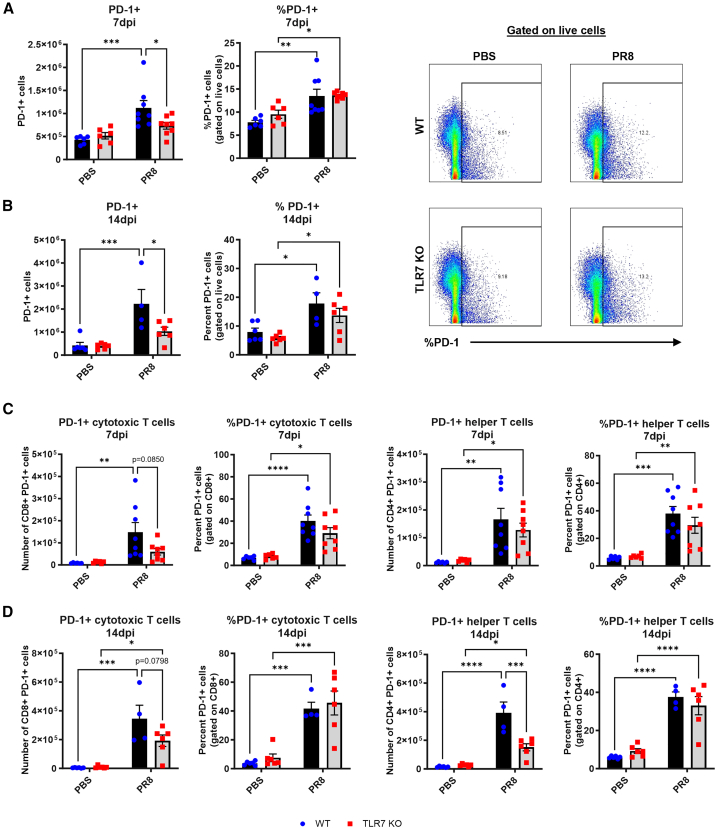
Reduced number of PD-1-expressing T cells in TLR7-KO mouse lungs following acute IAV infection WT C57BL/6 or TLR7-KO mice were intranasally infected with PR8 (50 PFUs) or mock infected with PBS. Flow cytometry was performed on lung tissue to measure the absolute numbers or frequencies of cells expressing PD-1 at (A) 7 or (B) 14 days post infection. PD-1 gating as a percentage of live cells at 7 dpi is shown. PD-1 expression was specifically measured on CD8^+^ cytotoxic and CD4^+^ helper subsets at (C) 7 or (D) 14 days post infection. Data are expressed as mean ± SEM (7 dpi: WT PBS *n* = 6, WT PR8 *n* = 8, TLR7-KO PBS *n* = 6, TLR7-KO PR8 *n* = 8; 14 dpi: WT PBS *n* = 6, WT PR8 *n* = 4, TLR7-KO PBS *n* = 6, TLR7-KO PR8 *n* = 6). Statistical analysis was conducted using two-way ANOVA test followed by Tukey’s post hoc test for multiple comparison test (∗*p* < 0.05, ∗∗*p* < 0.01, ∗∗∗*p* < 0.001, ∗∗∗∗*p* < 0.0001).

We next specifically examined changes in PD-1 expression in the lungs following IAV infection to determine whether TLR7 deficiency altered its regulation. In line with transcriptional data, the total number of PD-1+ lung cells increased significantly in WT but not TLR7-KO mice at both 7 and 14 dpi (Figures 2A and 2B). However, the frequency of PD-1+ cells increased similarly in both genotypes, indicating that the reduced total PD-1+ cell count in TLR7 KO lungs reflects impaired cell recruitment rather than a shift in cellular composition. This trend extended to T cell subsets. Infection increased the frequency and number of PD-1+ CD8^+^ cytotoxic and CD4^+^ helper T cells in WT lungs (Figures 2C and 2D). In TLR7-KO mice, frequencies were similar, but total cell numbers were significantly lower.

**Figure 3:**
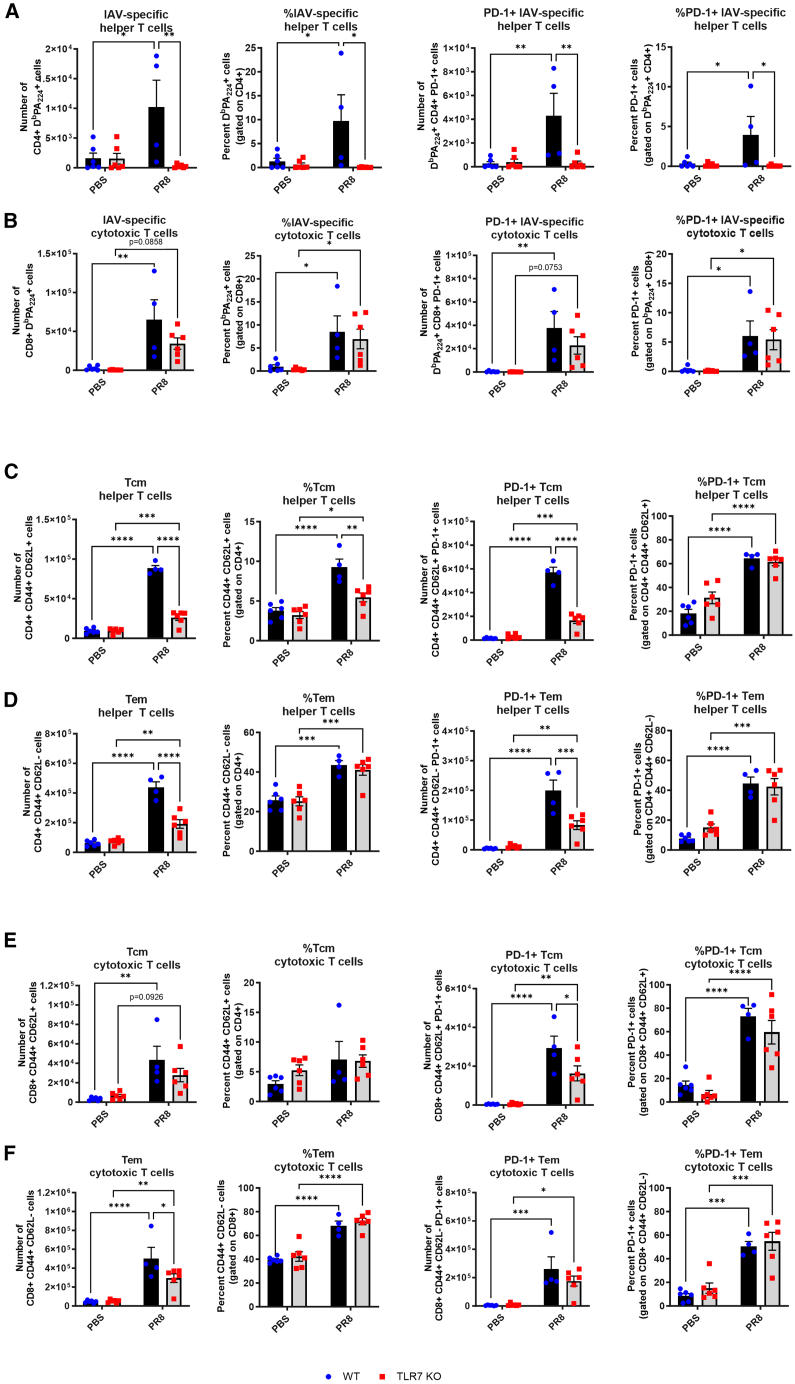
Reduced number of virus-specific or memory T cells in TLR7-KO mouse lungs following IAV infection WT C57BL/6 or TLR7-KO mice were intranasally infected with PR8 (50 PFUs) or mock infected with PBS. Flow cytometry was performed on lung tissue to measure the absolute numbers or frequencies of total cells or cells expressing PD-1 after 14 days post infection. IAV-specific (A) CD4^+^ helper and (B) CD8^+^ cytotoxic T cells bearing the DbPA224 epitope, (C) central memory or (D) effector memory CD4^+^ helper, and (E) central memory or (F) effector memory CD8^+^ cytotoxic T cells. Data are expressed as mean ± SEM (14 dpi: WT PBS *n* = 6, WT PR8 *n* = 4, TLR7-KO PBS *n* = 6, TLR7-KO PR8 *n* = 6). Statistical analysis was conducted using two-way ANOVA test followed by Tukey’s post hoc test for multiple comparison test (∗*p* < 0.05, ∗∗*p* < 0.01, ∗∗∗*p* < 0.001, ∗∗∗∗*p* < 0.0001).

To assess whether TLR7 influences PD-1 expression specifically on virus-specific T cells, we examined PA224-specific T cell responses at 14 dpi, a time point at which sustained PD-1 expression is associated with impaired T cell function.^13^ PA_224_+ CD4^+^ T cells were present in WT but were absent in TLR7-KO lungs, and this was mirrored by the absence of PD-1 expression on this subset (Figure 3A). In contrast, both genotypes had comparable numbers and PD-1 expression levels in PA_224_+ CD8^+^ T cells (Figure 3B). We also measured T cell memory subsets at 14 dpi. WT and TLR7-KO mice both showed infection-induced increases in CD4^+^ central (Tcm) and effector memory (Tem) T cells (Figures 3C and 3D). However, total memory T cell numbers, but not frequencies, were significantly reduced in TLR7-KO lungs. PD-1+ frequencies were similar between genotypes, except for a slight reduction in Tcm. Comparable results were observed for CD8^+^ Tcm and Tem subsets (Figures 3E and 3F).

Together, these data show that TLR7 deficiency does not significantly alter PD-1 expression on virus-specific or memory T cells. Rather, it impairs the overall recruitment of total and PD-1+ T cells into the lung during IAV infection.

### TLR7 deficiency impairs PD-L1 and PD-L2 responses in lung immune and non-immune cells during IAV infection

**Figure 4:**
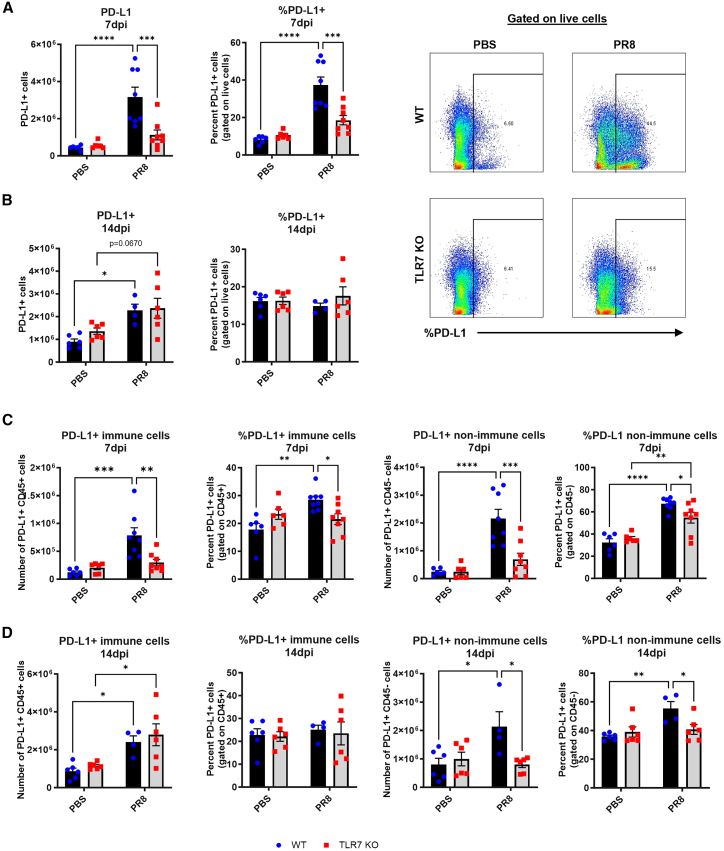
Reduced number of lung cells expressing PD-L1 in TLR7-KO mouse lungs following IAV infection WT C57BL/6 or TLR7-KO mice were intranasally infected with PR8 (50 PFUs) or mock infected with PBS. Flow cytometry was performed on lung tissue to measure the absolute numbers or frequencies of cells expressing PD-L1 at (A) 7 or (B) 14 days post infection. PD-L1 gating as a percentage of live cells at 7 dpi is shown. PD-L1 expression was specifically measured on CD45^−^non-immune and CD45^+^ immune cells at (C) 7 or (D) 14 days post infection. Data are expressed as mean ± SEM (7 dpi: WT PBS *n* = 6, WT PR8 *n* = 8, TLR7-KO PBS *n* = 6, TLR7-KO PR8 *n* = 8; 14 dpi: WT PBS *n* = 6, WT PR8 *n* = 4, TLR7-KO PBS *n* = 6, TLR7-KO PR8 *n* = 6). Statistical analysis was conducted using two-way ANOVA test followed by Tukey’s post hoc test for multiple comparison test (∗*p* < 0.05, ∗∗*p* < 0.01, ∗∗∗*p* < 0.001, ∗∗∗∗*p* < 0.0001).

We next examined how TLR7 signaling influences the expression of the PD-1 ligands, PD-L1 and PD-L2, in the lungs following IAV infection. At 7 dpi, WT mice had significantly higher numbers of PD-L1+ lung cells than TLR7-KO mice, consistent with elevated *CD274* transcript levels (Figure 4A). Unlike PD-1, where cellular frequencies were unchanged across genotypes, PD-L1+ cell frequency was also significantly lower in TLR7-KO lungs, suggesting that TLR7 regulates both recruitment and expression of PD-L1. By 14 dpi, PD-L1 expression remained elevated in both genotypes, with no significant difference (Figure 4B).

We then analyzed PD-L1 expression within immune (CD45^+^) and non-immune (CD45^−^) lung cells. At 7 dpi, both the number and frequency of PD-L1+ immune and non-immune cells were significantly higher in WT mice compared to TLR7 KO (Figure 4C). At 14 dpi, PD-L1 expression in immune cells had equalized between genotypes, but both the number and frequency of PD-L1+ non-immune cells remained significantly lower in TLR7-KO mice (Figure 4D), indicating delayed or incomplete induction in epithelial or stromal compartments.

**Figure 5:**
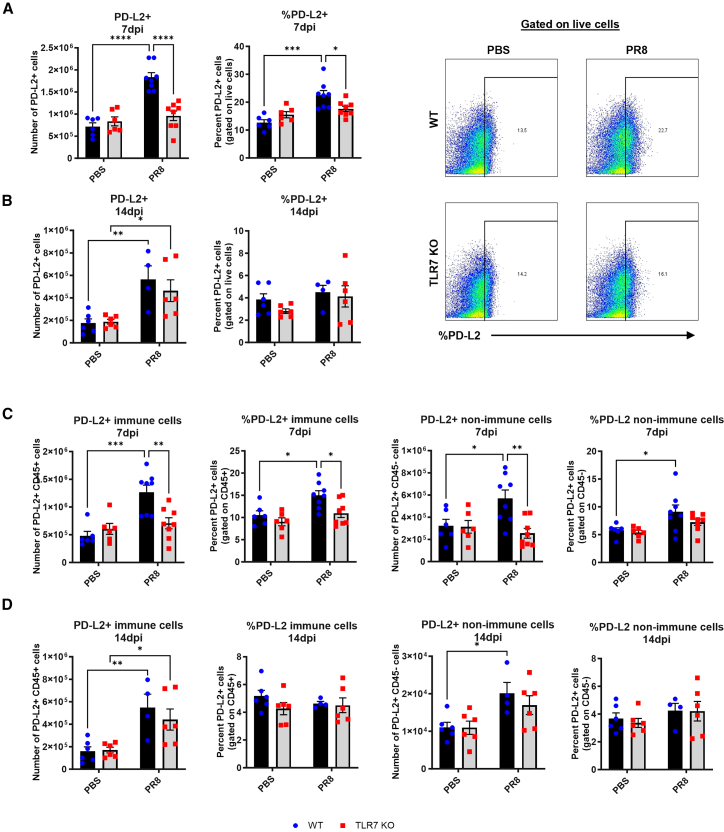
Reduced number of lung cells expressing PD-L2 in TLR7-KO mouse lungs following IAV infection WT C57BL/6 or TLR7-KO mice were intranasally infected with PR8 (50 PFUs) or mock infected with PBS. Flow cytometry was performed on lung tissue to measure the absolute numbers or frequencies of cells expressing PD-L2 at (A) 7 or (B) 14 days post infection. PD-L2 gating as a percentage of live cells at 7 dpi is shown. PD-L2 expression was specifically measured on CD45^−^non-immune and CD45^+^ immune cells at (C) 7 or (D) 14 days post infection. Data are expressed as mean ± SEM (7 dpi: WT PBS *n* = 6, WT PR8 *n* = 8, TLR7-KO PBS *n* = 6, TLR7-KO PR8 *n* = 8; 14 dpi: WT PBS *n* = 6, WT PR8 *n* = 4, TLR7-KO PBS *n* = 6, TLR7-KO PR8 *n* = 6). Statistical analysis was conducted using two-way ANOVA test followed by Tukey’s post hoc test for multiple comparison test (∗*p* < 0.05, ∗∗*p* < 0.01, ∗∗∗*p* < 0.001, ∗∗∗∗*p* < 0.0001).

A similar pattern was observed for PD-L2. At 7 dpi, the total number and frequency of PD-L2+ lung cells were significantly reduced in TLR7-KO mice (Figure 5A), while by 14 dpi, levels had normalized across genotypes (Figure 5B). This early defect was seen in both immune and non-immune cell compartments at 7 dpi (Figure 5C), but resolved by 14 dpi (Figure 5D). To determine whether differences in PD-L expression were due to changes in immune cell composition, we examined expression of PD-L1 and PD-L2 among T cell, B cell, and myeloid cells (Figure S2). Ligand expression increased in CD11b+CD11c+ cells after infection, while TLR7 deficiency did not significantly shift the proportion of these subsets. This indicates that the reduced PD-L1 and PD-L2 expression was not due to altered immune cell recruitment or phenotypic skewing.

Together, this analysis reveals that TLR7 deficiency impairs the induction of PD-L1 and PD-L2 during early IAV infection, across both immune and non-immune lung cells. Rather than altering the cellular makeup of the lung, TLR7 deficiency reduces the recruitment and distribution of PD-L-expressing cells in the lung, potentially impairing checkpoint-mediated immunoregulation.

### TLR7 activation regulates PD-L1, but not PD-1, expression in a cytokine-dependent manner

To examine the impact of TLR7 on PD-1 and PD-L1 expression in the lung, we calculated the PD-1:PD-L1 ratio across time points. In WT mice, a PD-L1-dominant profile was observed at 7 dpi, which shifted toward PD-1 predominance by 14 dpi (Figure S3), consistent with evolving immune activation and exhaustion phases. This shift was absent in TLR7-KO mice, whose ratios remained similar to those of uninfected controls throughout infection, suggesting that TLR7 drives this temporal checkpoint transition.

**Figure 6:**
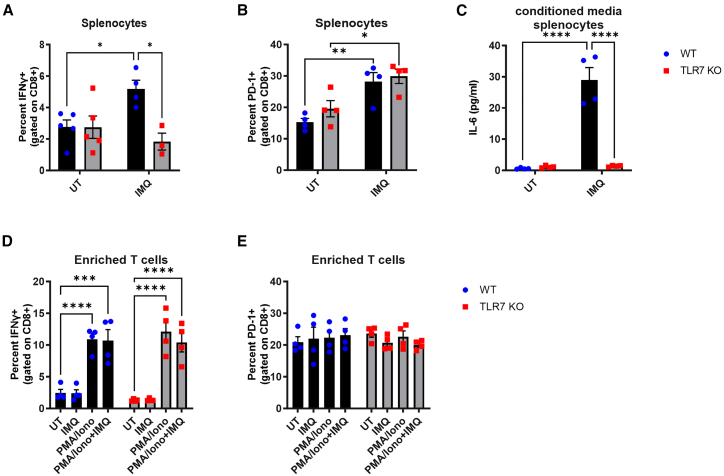
TLR7 stimulation does not alter PD-1 expression on T cells Splenocytes from naive WT C57BL/6 or TLR7-KO mice were exposed *ex vivo* for 24 h to imiquimod (IMQ, 10 μg/mL). CD8^+^ T cells were gated and staining for (A) intracellular IFN-γ, or (B) surface PD-1 was determined by flow cytometry. (C) Secretion of IL-6 into the culture media was measured by ELISA. T cells isolated from naive splenocytes were also exposed to IMQ (10 μg/mL), phorbol 12-myristate 13-acetate (PMA, 1 μM) plus ionomycin (Iono, 0.5 μM), or a combination of both treatments for 24 h. Purified CD8^+^ T cells were gated and stained for (D) intracellular IFN-γ or (E) surface PD-1 expression. Data are expressed as mean ± SEM, *n* = 4–5 independent repeats. Statistical analysis was conducted using two-way ANOVA test followed by Tukey’s or Sidak’s post hoc test for multiple comparison test (∗*p* < 0.05, ∗∗*p* < 0.01, ∗∗∗*p* < 0.001, ∗∗∗∗*p* < 0.0001).

To determine whether TLR7 directly affects PD-1 or PD-L1 expression on specific cells, we performed *ex vivo* stimulation experiments using the TLR7 agonist imiquimod (IMQ). In naive splenocyte cultures from WT mice, IMQ induced interferon γ (IFN-γ) production in CD8^+^ T cells, while TLR7-KO cells failed to respond (Figure 6A). Despite this differential activation, PD-1 expression increased similarly in both genotypes (Figure 6B), suggesting that PD-1 upregulation is not dependent on TLR7 signaling or IFN-γ production. Interleukin 6 (IL-6) production was also absent in TLR7-KO splenocytes (Figure 6C), confirming a defective cytokine response in these cells to TLR7 stimulation.

To isolate potential T cell-intrinsic effects, we treated purified T cells with IMQ or phorbol 12-myristate 13-acetate (PMA)/ionomycin. PMA/ionomycin, but not IMQ, induced intracellular IFN-γ accumulation; however, neither treatment changed PD-1 expression (Figures 6D and 6E). This indicates that TLR7 stimulation alone is insufficient to directly drive activation or PD-1 expression in T cells and likely requires co-stimulation or paracrine signaling from other cell types.

**Figure 7:**
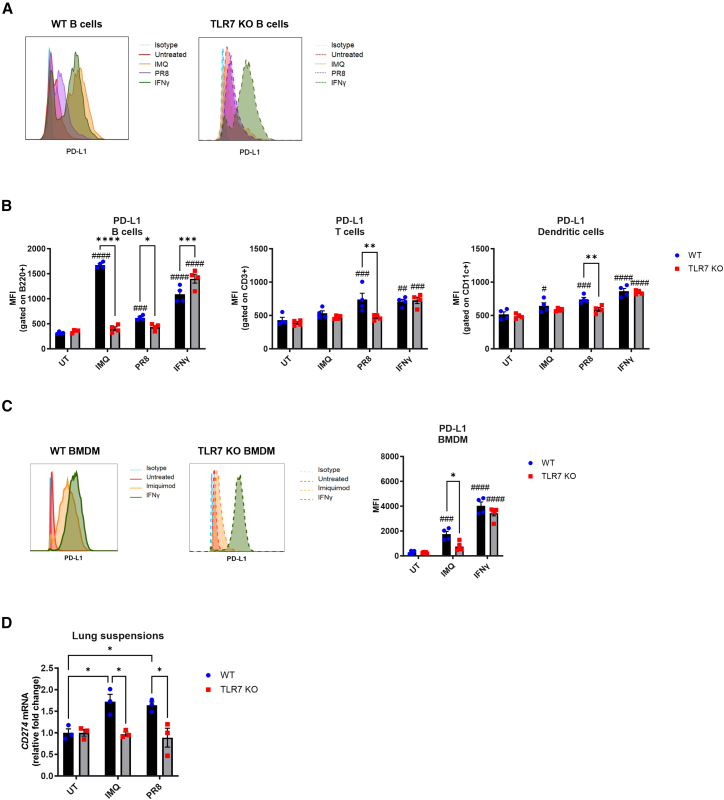
TLR7 stimulation boosts PD-L1 expression Splenocytes from naive WT C57BL/6 or TLR7-KO mice were exposed *ex vivo* for 24 h to imiquimod (IMQ, 10 μg/mL), PR8 virus (MOI of 1), or recombinant IFN-γ (0.1 ng/mL). Cells were then stained for PD-L1 and measured by flow cytometry. (A) Representative histograms of PD-L1 expression are shown. (B) Mean fluorescent intensities (MFIs) of B cells (B220+), T cells (CD3^+^), or dendritic cells (CD11c^+^) expressing PD-L1 are shown. (C) Bone marrow-derived macrophages (BMDMs) were treated with IMQ (10 μg/mL) or IFN-γ (0.1 ng/mL) for 24 h and PD-L1 surface expression determined by flow cytometry. (D) Single cell lung suspensions from naive WT C57BL/6 or TLR7-KO mice were *ex vivo* exposed to imiquimod (IMQ, 10 μg/mL) or PR8 virus (MOI of 1) for 24 h. Gene expression of PD-L1 (CD274 mRNA) was measured and expressed relative to RPS18 housekeeping as a fold-change above non-treated controls of each mouse genotype. Data are expressed as mean ± SEM, *n* = 3–4 independent repeats. Statistical analysis was conducted using two-way ANOVA test followed by multiple comparison using Tukey’s post hoc test to compare differences between treatments and each genotype (∗*p* < 0.05, ∗∗*p* < 0.01, ∗∗∗*p* < 0.001, ∗∗∗∗*p* < 0.0001) or Sidak’s post hoc test to compare differences between untreated groups for each respective genotype (#*p* < 0.05, ##*p* < 0.01, ###*p* < 0.001, ####*p* < 0.0001).

In contrast, TLR7 activation had a clear impact on PD-L1 expression. Stimulation of splenocytes with either IMQ or PR8 virus significantly increased PD-L1 expression in B cells from WT but not TLR7-KO mice (Figures 7A and 7B). PR8 also elevated PD-L1 expression on CD3^+^ T cells and CD11c+ dendritic cells in a TLR7-dependent manner. To test whether this was a cell-intrinsic effect, we treated WT and TLR7-KO bone marrow-derived macrophages (BMDMs) with IMQ or recombinant IFN-γ. While IFN-γ induced PD-L1 in both genotypes, IMQ upregulated PD-L1 only in WT BMDMs (Figure 7C), confirming that TLR7 directly mediates this effect. Similarly, lung cell suspensions from WT but not TLR7-KO mice showed increased *CD274* (PD-L1) transcription after IMQ or PR8 exposure (Figure 7D), confirming that TLR7 mediates PD-L1 induction in lung-resident cells.

**Figure 8:**
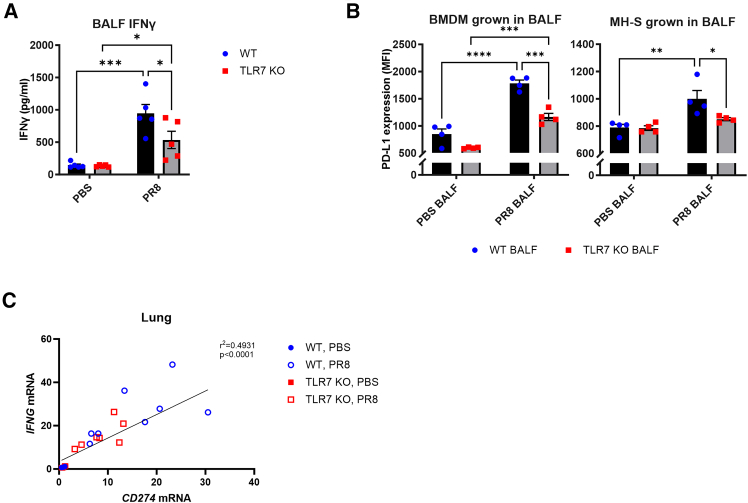
Soluble factors in WT-infected BALF promote PD-L1 upregulation on target cells (A) IFN-γ protein levels were measured in the bronchioalveolar fluid (BALF) of WT C57BL/6 or TLR7-KO mice infected with PR8 (50 PFUs) or mock infected with PBS after 7 days. (B) BMDM or alveolar MH-S macrophages were grown in BALF and surface expression of PD-L1 measured after 24 h by flow cytometry. (C) Simple linear regression tests were performed on lung mRNA expression of IFNG and CD274 from experimental mice. Data are expressed as mean ± SEM, *n* = 4–5 independent cell experiments, or *n* = 5–8 mice per experimental group. Statistical analysis was conducted using two-way ANOVA test followed by Tukey’s post hoc test for multiple comparison test (∗*p* < 0.05, ∗∗*p* < 0.01, ∗∗∗*p* < 0.001, ∗∗∗∗*p* < 0.0001).

Finally, to determine whether the inflammatory environment contributes to PD-L1 regulation *in vivo*, we collected bronchioalveolar lavage fluid (BALF) from PR8-infected WT and TLR7-KO mice at 7 dpi and used it to culture and stimulate macrophages. BALF from TLR7-KO mice contained significantly lower levels of IFN-γ (Figure 8A), consistent with reduced proinflammatory cytokine production in their airways. When BMDMs or alveolar macrophages (MH-S) were cultured in BALF from infected WT mice, PD-L1 surface expression was significantly upregulated (Figure 8B), whereas BALF from TLR7-KO mice showed significantly lower PD-L1 expression. Additionally, a strong correlation was observed between *IFNG* and *CD274* mRNA expression in infected lungs (Figure 8C), supporting a role for IFN-γ in driving PD-L1 upregulation in the airway environment.

Collectively, these findings demonstrate that TLR7 activation promotes PD-L1 expression in both immune and lung-resident cells through a combination of direct and cytokine-mediated mechanisms, such as via IFN-γ. In contrast, PD-1 expression on T cells is not directly regulated by TLR7 activity and appears uncoupled from TLR7-mediated activation pathways.

## Discussion

This study reveals an underappreciated mechanism by which TLR7 modulates immune checkpoint dynamics during IAV infection, identifying it as a key upstream regulator of the PD-1/PD-L axis. While TLR7 is well established as a critical sensor of single-stranded viral RNA that promotes antiviral and proinflammatory responses, our findings demonstrate that it also contributes to T cell regulation by driving the expression of PD-L1 and PD-L2 in both immune and non-immune lung compartments. This TLR7-dependent increase in ligand availability enhances PD-1 engagement, impairing effector T cell function and promoting transcriptional features of T cell exhaustion during key stages of the immune response. These results position TLR7 as a link between innate antiviral sensing and adaptive immune suppression, with implications for both infection outcomes and broader immune regulation in the lung.

Transcriptomic analysis revealed a stronger T cell exhaustion signature in the lungs of WT mice compared to TLR7-KO mice, particularly at later stages of infection. In the acute phase, WT mice showed elevated expression of T cell activation markers (*IFNG*, *IL2*, and *TBX21*), indicating a more rapid immune activation in the presence of TLR7. However, concurrent upregulation of *TOX* suggested an early onset of exhaustion, which persisted through 14 dpi. By this time point, WT mice exhibited a high PD-1:PD-L1 ratio, indicative of sustained PD-1-mediated signaling and potential progression toward exhaustion. This pattern suggests that PD-1 expression at this later stage reflects persistent inhibitory signaling rather than the transient, activation-induced PD-1 upregulation typically seen earlier during infection. Chronic elevation of PD-1 following IAV infection is associated with poor memory T cell responses.^13^ Importantly, although PD-1 expression on central and effector memory T cells was comparable between genotypes, exhaustion- and checkpoint-related genes appeared more dominant in WT mice, likely due to greater antigenic and inflammatory stimulation. Despite the attenuated inflammatory response, TLR7-KO mice were still capable of mounting acute T cell activation and effectively cleared the virus, as evidenced by their lower lung viral load. This suggests that a more moderate T cell response in the absence of TLR7 is sufficient for viral control. Alternatively, our previous work indicates that TLR7 activity in the upper respiratory tract (URT) promotes a more robust innate and adaptive immune response in the lower respiratory tract.^34^ Therefore, the TLR7-dependent checkpoint regulation observed in the lung may, at least in part, be influenced by immune responses initiated in the URT. Future studies using direct intratracheal administration of virus to the lung, thereby bypassing the URT, could further delineate lung-specific effects of TLR7 on T cell regulation. Nonetheless, TLR7 is essential for optimal memory T cell and antibody responses to IAV.^42^^,^^43^ Thus, while viral clearance can indeed occur in TLR7-deficient hosts, likely due to compensatory antiviral responses by other pattern recognition receptors such as TLR3 or Retinoic acid-inducible gene I (RIG-I), long-term immune protection appears compromised. Despite the potential redundancy and crosstalk among TLR pathways in IFN and nuclear factor κB (NF-κB) signaling,^44^ the checkpoint and T cell exhaustion phenotypes described in this study are unique to TLR7-driven inflammation during IAV infection.

Mechanistically, our data show that TLR7 selectively regulates PD-L1 and PD-L2 expression. TLR7-deficiency resulted in reduced recruitment and surface expression of PD-L1 and PD-L2 on both immune and non-immune lung cells, while PD-1 T cell recruitment, but not PD-1 expression itself, was impaired. Although PD-L expression on T cells, B cells, dendritic cells, and myeloid cells remained unchanged, overall PD-L levels were reduced. Considering the high TLR7 expression in these cells, future studies should investigate how TLR7-driven modulation of PD-L during IAV infection may influence antigen presentation and antibody responses. *In vitro*, PR8 infection of immune cells or TLR7 agonist stimulation induced PD-L1 expression, in a TLR7-dependent manner, supporting the conclusion that TLR7-mediated viral sensing drives ligand upregulation, particularly in immune cell populations. This contrasts with epithelial cells, which express minimal TLR7^45^ and upregulate PD-L1 through Src homology region 2 domain-containing phosphatase 2 (SHP2) and Janus kinase (JAK)/signal transducers and activators of transcription (STAT) signaling during IAV infection.^19^ Consistent with prior studies, we show that IMQ-stimulated splenocytes and macrophages from WT mice upregulate PD-L1, while TLR7-KO cells fail to respond. This aligns with previous work demonstrating that TLR-driven PD-L1 upregulation is mediated via STAT3 and sustained by IL-6 and IL-10 signaling.^46^^,^^47^ In line with this, TLR7-KO splenocytes produced less IL-6 and exhibited diminished PD-L1 expression. Moreover, IFN-γ, a potent PD-L1 inducer via JAK/STAT pathways,^48^ was reduced in TLR7-KO BALF and correlated with PD-L1 levels in lung tissue. Other inflammatory mediators, including IL-6, IL-10, and type I interferons, which can also regulate PD-L1, were likewise decreased in the airways and lungs of IAV-infected TLR7-KO mice.^31^^,^^34^ Macrophages cultured in BALF from infected WT mice upregulated PD-L1, whereas those cultured with BALF from TLR7-KO mice showed impaired induction. Together, these findings exemplify that TLR7-mediated inflammation promotes PD-L1 expression through both direct sensing and cytokine-driven paracrine signaling, and that the overall reduction in inflammatory cues in TLR7-deficient cells contributes to the attenuated PD-L1/PD-1 signaling observed during infection.

Elevated PD-L1 expression during infection may help protect lung tissue by limiting cytotoxic T cell-mediated immunopathology. However, this immunosuppressive shift can allow virus-infected cells to evade immune clearance and may lead to persistent exhaustion, particularly when PD-1 levels are chronically elevated.^49^^,^^50^ Supporting this, Rutigliano et al.^14^ observed that highly inflammatory PR8 infections induced more PD-1 expression on IAV-specific T cells and less effector function, when compared to the less pathogenic Hk-X31 strain. In our PR8 model, TLR7 did not directly control PD-1 expression on virus-specific or memory T cells. Instead, TLR7-driven inflammation enhanced early T cell activation and potentially promoted exhaustion through increased PD-L1/PD-L2 engagement. This may be further exacerbated by higher viral loads in WT mice, as persistent antigen exposure can sustain PD-1 expression and drive T cell exhaustion.^51^ Our findings indicate that TLR7 deficiency itself results in lower PD-L1 induction during infection, effectively mimicking aspects of PD-L1 blockade. Indeed, PD-L1 blockade during IAV infection enhances IFN-γ production and cytolytic function of virus-specific CD8^+^ T cells, reduces lung viral titers, and mitigates disease morbidity.^15^ Interestingly, virus-induced airway dysfunction has also been reported upon PD-L antibody blockade,^13^ suggesting a protective role for PD-L expression in maintaining lung function. In our previous study, TLR7 deficiency led to IAV-induced late-stage airway hyper-responsiveness correlating with CD8^+^ T cell accumulation.^31^ Given the reduced PD-L expression in TLR7-KO lungs, it is plausible that TLR7 activity helps sustain PD-L levels to limit T cell-driven chronic pathology. While further investigation is needed, these findings highlight the dual, context-dependent role of TLR7 in balancing immune protection and immunopathology during infection.

Our findings also have implications for lung tumor immunosurveillance. Epidemiological and experimental evidence suggest that influenza infection can impair antitumor immunity, accelerating non-small cell lung cancer progression by upregulating PD-1 on T cells and PD-L1 on tumor cells.^52^^,^^53^ Similarly, in melanoma models, IAV redirected cytotoxic T cells toward infected lung tissue, limiting tumor clearance.^54^ Conversely, prior IAV exposure can enhance antitumor immunity and improve responses to checkpoint blockade therapy in several cancer models.^55^^,^^56^ These dual effects highlight how TLR7-driven inflammation may transiently remodel the lung microenvironment to suppress immunity. Our results support the concept that modulating TLR7 activity could help restore immune balance and potentially synergize with PD-1/PD-L1 blockade to enhance antitumor defense.^25^^,^^27^

In summary, our results identify TLR7 as a central regulator of PD-L1-mediated immune suppression during IAV infection. While TLR7 activation enhances early T cell responses, it also drives PD-L1 upregulation, which may ultimately impair effector T cell function by promoting both early and sustained T cell exhaustion. This PD-L1-mediated suppression likely dampens, but does not fully prevent, the effects of the TLR7-driven “cytokine storm,” thereby increasing the risk of immune-mediated pathology. Targeting the TLR7-PD-L1 axis may therefore offer a strategy to preserve antiviral immunity, reduce immunopathology, and potentially improve outcomes in both infectious and neoplastic diseases of the lung.

### Limitations of the study

Experiments were conducted exclusively in male mice; given known sex differences in antiviral immunity and X-linked TLR7 expression, validation in females is warranted. Analyses focused on acute and early-to-mid resolution phases of IAV infection, and later time points may be required to determine whether TLR7-dependent checkpoint regulation contributes to persistent T cell exhaustion and affects recall responses. While global exhaustion signatures were examined in the lungs, more detailed T cell-intrinsic analyses of virus-specific subsets would strengthen mechanistic conclusions. In addition, the effects of immune checkpoint blockade were not directly tested; assessing PD-1 or PD-L1 inhibition, alone or in combination with TLR7 modulation, would help establish therapeutic relevance.

## Resource availability

### Lead contact

Further information and requests for resources and reagents should be directed to and will be fulfilled by the lead contact, Stavros Selemidis (stavros.selemidis@rmit.edu.au).

### Materials availability

This study did not generate new unique reagents.

### Data and code availability

• All data reported in this paper will be shared by the lead contact upon request.
• This paper does not report original code.
• Any additional information required to reanalyze the data reported in this paper is available from the lead contact upon request.

## Acknowledgments

This work was supported by the 10.13039/501100000925National Health and Medical Research Council of Australia (NHMRC project IDs: 1122506, 1128276, and 2002948).

## Author contributions

Conceptualization, M.A.M., J.J.O’L., D.A.B., and S.S.; methodology and writing – original draft, M.A.M. and S.S.; investigation, M.A.M., S.L., and F.L.; writing – review and editing, M.A.M., S.L., F.L., J.J.O’L., D.A.B., and S.S.; funding acquisition, J.J.O’L., D.A.B., and S.S.; resources and supervision, S.S.

## Declaration of interests

The authors declare no competing interests.

## STAR★Methods

### Key resources table

**Table:** 

REAGENT or RESOURCE	SOURCE	IDENTIFIER
**Antibodies**		

Rat anti-mouse CD45-Alexa Fluor 700 (clone 30-F11)	Biolegend	Cat#103128, RRID:AB_493715
Rat anti-mouse CD45-BV605 (clone 30-F11)	Biolegend	Cat#103155, RRID:AB_2650656
Rat anti-mouse CD3-APC (clone 17A2)	Thermo Fisher Scientific	Cat#17-0032-82; RRID:AB_10597589
Rat anti-mouse CD3-PerCP (clone 145-2C11)	Biolegend	Cat#100326, RRID:AB_893317
Rat anti-mouse CD4-BV605 (clone RM4-5)	Biolegend	Cat#100548, RRID:AB_2563054
Rat anti-mouse CD8a-PacificBlue (clone 53-6.7)	Biolegend	Cat#100725; RRID: AB_493425
Rat anti-mouse CD44-FITC (clone IM7)	BD Biosciences	Cat# 553133, RRID:AB_2076224
Rat anti-mouse CD62L-PerCP (clone MEL-14)	Biolegend	Cat#104430, RRID:AB_2187124
Rat anti-mouse PD-1-APC-Cy7 (clone M1/70)	Biolegend	Cat#135224, RRID:AB_2563523
Rat anti-mouse PD-1-PE (clone 29F.1A12)	Biolegend	Cat#135206, RRID:AB_1877231
Rat anti-mouse PD-L1-PE (clone 10F.9G2)	Biolegend	Cat#124308, RRID:AB_2073556
Rat anti-mouse PD-L2-PerCP-Cy5.5 (clone TY25)	Biolegend	Cat#107218, RRID:AB_2728126
Rat anti-mouse CD11b-APC-Cy7 (clone M1/70)	Biolegend	Cat#101226, RRID:AB_830642
Rat anti-mouse CD11c-PE-Cy7 (clone N418)	Thermo Fisher Scientific	Cat#25-0114-82, RRID:AB_469590
Rat anti-mouse B220-FITC (clone RA3-6B2)	Biolegend	Cat#103206, RRID:AB_312991
Rat anti-mouse CD16/32 (clone 2.4G2)	Biolegend	Cat#101301, RRID:AB_312800
Rat anti-mouse IFNγ-AF700 (clone XMG1.2)	Biolegend	Cat#505824, RRID:AB_2561300
Rat anti-mouse IgG2a-PE isotype (clone RTK2758)	Biolegend	Cat#400508, RRID:AB_326530

**Bacterial and virus strains**		

PR8/A virus (H1N1 strain)	Patrick Reading, The Peter Doherty Institute Melbourne	–

**Chemicals, peptides, and recombinant proteins**		

DbPA224-APC peptide	Innate Immunity and Anti-Viral Immunity Laboratory, Department of Microbiology and Immunology, University of Melbourne	–
Liberase	Merck	Cat#5401119001
Cytofix/Cytoperm Fixation/Permeabilization Kit	BD Biosciences	Cat#554714
LIVE/DEAD Fixable Aqua Dead Cell Stain Kit	Invitrogen	Cat#L34966
Imiquimod	Invivogen	Cat#tlrl-imqs-1
Mouse IFN-gamma Recombinant Protein	Peprotech	Cat#315-05-100UG
Human IL-2 Recombinant Protein	Roche	Cat#11011456001
Phorbol 12-myristate 13-acetate	Sigma	Cat#P8139
Ionomycin from Streptomyces Conglobatus	Sigma	Cat#I9657

**Critical commercial assays**		

Mouse IFN-gamma DuoSet ELISA	R&D Systems	Cat#DY485
Mouse IL-6 DuoSet ELISA	R&D Systems	Cat#DY406
Pan T cell Isolation Kit II, mouse	Miltenyi Biotec	Cat#130-095-130

**Experimental models: Cell lines**		

Immortalized murine bone marrow-derived macrophages	Ashley Mansell, Hudson Institute of Medical Research Monash University	–
MH-S	ATCC	Cat#CRL-2019, RRID:CVCL_3855

**Experimental models: Organisms/strains**		

Mouse: Wild type: C57Bl/6J	Animal Resources Center (Western Australia, Australia)	RRID:IMSR_JAX:000664
Mouse: TLR7 KO: B6.129S1-Tlr7tm1Flv/J	The Jackson Laboratory (Maine, USA)	RRID:IMSR_JAX:008380

**Oligonucleotides**		

Primer for FAM-conjugated Influenza A Polymerase: Forward: 5′-CGGTCCAAATTCCTGCTGA-3′ Reverse: 5′-CATTGGGTTCCTTCCATCCA-3′	Life Technologies	–
CD274 (Mm00452054_m1)	Life Technologies	Cat#4331182
CD69 (Mm01183378_m1)	Life Technologies	Cat#4331182
CTLA4 (Mm00486849_m1)	Life Technologies	Cat#4331182
IFNG (Mm00436450_m1)	Life Technologies	Cat#4331182
IL2 (Mm00434256_m1)	Life Technologies	Cat#4331182
IL12B (Mm01288989_m1)	Life Technologies	Cat#4331182
IRF7 (Mm00516793_g1)	Life Technologies	Cat#4331182
NFATC1 (Mm01265944_m1)	Life Technologies	Cat#4331182
PDCD1 (Mm00435532_m1)	Life Technologies	Cat#4331182
PDCD1LG2 (Mm00451734_m1)	Life Technologies	Cat#4331182
PRDM1 (Mm00476128_m1)	Life Technologies	Cat#4331182
RELA (Mm00501346_m1)	Life Technologies	Cat#4331182
RPS18 (Mm02601777_g1)	Life Technologies	Cat#4331182
TBX21 (Mm00450960_m1)	Life Technologies	Cat#4331182
TIGIT (Mm03807522_m1)	Life Technologies	Cat#4331182
TLR7 (Mm00446590_m1)	Life Technologies	Cat#4331182
TOX (Mm00455231_m1)	Life Technologies	Cat#4331182

**Software and algorithms**		

GraphPad Prism (Version 10.0)	GraphPad Prism	RRID:SCR_002798
FlowJo (Version 10.0)	FlowJo	RRID:SCR_008520

### Experimental model and participant details

#### Animals - mice

Male wild type (C57BL/6J) mice were obtained from the Animal Resources Center (Western Australia, Australia). Homozygous TLR7 knockout mice (B6.129S1-Tlr7tm1Flv/J, JAX stock #008380) were obtained from The Jackson Laboratory (Maine, USA)^57^ and bred in-house at the RMIT University animal research facility (Bundoora, Australia). Mice were housed under standard conditions (12 h light/12 h dark cycle) with *ad libitum* access to food and water. Mice were randomly assigned to experimental groups. Only male mice were used in this study, as they mount a more vigorous immune response to IAV infection than female mice.^58^ All animal experiments were approved by the Royal Melbourne Institute of Technology University (RMIT) Animal Ethics Committee (Ethics number 23328) and in compliance with the guidelines of the National Health and Medical Research Council (NHMRC) of Australia on animal experimentation.

#### Cell lines and primary cell cultures

Primary splenocytes or lung suspensions from male WT (C57BL/6J) and TLR7 KO (B6.129S1-Tlr7tm1Flv/J) mice were cultured in complete RPMI-1640 media (containing with Glutamax, 10% fetal bovine serum (FBS) and 1% penicillin-streptomycin) or complete DMEM media (containing with L-glutamine, 4500 mg/mL glucose, sodium pyruvate 110 mg/L, 10% FBS, and 1% penicillin-streptomycin), respectively. Immortalized alveolar MH-S macrophages were originally obtained from a 7-week-old male mouse (ATCC, CRL-2019), and cultured in complete RPMI-1640 media. Immortalized bone marrow-derived macrophages (BMDMs) were originally obtained from an 8-week-old male mouse (courtesy of Ashley Mansell of the Hudson Institute of Medical Research Monash University) and cultured in complete DMEM media. All cells were grown under standard conditions at 37 °C in air supplemented with 5% CO_2_. Independent cell line authentication or mycoplasma testing was not performed for this study.

### Method details

#### IAV infections

8–14-week-old mice were anesthetized by isoflurane inhalation and infected intranasally with 50 plaque-forming units (PFUs) of PR8 (H1N1 strain) in 35 μL of phosphate buffered saline (PBS). Control animals received PBS alone. Mice were then weighed and monitored daily. Mice were euthanized by injection (i.p) of a mixture of ketamine (180 mg/kg) and xylazine (32 mg/kg) at experimental endpoints.

#### Immune cell phenotyping by flow cytometry

Whole lung was finely minced using scissors and then enzymatically digested using 1% Liberase (Sigma) for 45 min at 37 °C shaking at 700 rpm. Tissues were homogenized then single cell suspensions prepared by straining through a 40 μm strainer. After lysing the red blood cells with ammonium-chloride-potassium (ACK) lysis buffer, cells were stained with cocktail mixtures of fluorescent-labelled anti-mouse antibodies diluted in FACS buffer (PBS with 2.5% fetal bovine serum; FBS) for 30 min on ice. The following Biolegend antibodies were used (unless stated otherwise): CD45-AF700 (clone 30-F11), CD45-BV605 (clone 20-F11), CD3-APC (clone 17A2, eBioscience), CD4-BV605 (clone RM4-5), CD8a-PacificBlue (clone 53-6.7), CD44-FITC (clone IM7, BD Bioscience), CD62L-PerCP (clone MEL-14), PD-1-APC-Cy7 (clone 29F.1A12), PD-L1-PE (clone 10F.9G2), PD-L2-PerCP-Cy5.5 (clone TY25), CD11b-APC-Cy7 (clone M1/70), CD11c-PE-Cy7 (clone N418; eBioscience), and B220-FITC (clone RA3-6B2). Tetramer staining of virus-specific CD8^+^ T cells was performed using an APC conjugated DbPA224 peptide that was synthesized by the Innate Immunity and Anti-Viral Immunity Laboratory in the Department of Microbiology and Immunology, University of Melbourne. CD16/32 (clone 2.4G2) and LIVE/DEAD Fixable Aqua Dead Cell Stain Kit (Invitrogen) were contained within each antibody cocktail mixture to block of Fc-mediated adherence of the antibodies and to exclude dead cells, respectively.

For *ex vivo* cultures, the following Biolegend antibodies were used for surface staining: CD3-PerCP (clone 145-2C11), CD8-PacificBlue (clone 53-6.7), CD11c-PE-Cy7 (clone N418; eBioscience), B220-FITC (clone RA3-6B2), PD-1-PE (clone 29F.1A12), PD-L1-PE (clone 10F.9G2) or IgG2a-PE isotype (clone RTK2758). In some experiments, GolgiPlug (1:1000) and GolgiStop (1:2000) was added to the cells for the final 5 h of treatment to facilitate intracellular IFNγ cytokine detection. After initially staining cells for surface markers, cells were fixed and permeabilized using the BD Cytofix/Cytoperm Fixation/Permeabilization Kit (BD Biosciences) and stained with IFNγ-AF700 (clone XMG1.2; Biolegend).

All samples were processed on a BD LSRFortessaTM X-20 flow cytometry analyzer with DIVA software (Becton Dickinson Bioscience, USA) and data analyzed using FlowJo software (Tree Star, Inc.). Cells were analyzed as a percentage of live cells (Amcyan negative populations) or parent population, and as absolute numbers relative to amount of lung tissue (g) processed. Representative gating strategies are shown in Figures S4 and S5.

#### RNA extraction and qPCR

Lungs were harvested for RNA extraction using the RNeasy Mini kit (Qiagen, USA), as per manufacturer’s instructions. RNA sample concentration and quality were measured using the Nanodrop one Spectrophotometer (Thermofisher Scientific, USA). The cDNA synthesis was performed on 1–2 μg of total RNA using the High-Capacity cDNA Reverse Transcription Kit (Applied Biosystems, Foster City, CA, USA) according to the following settings: 25 °C for 10 min, 37 °C for 120 min, 85 °C for 5 min. Quantitative polymerase chain reaction was carried out using the TaqMan Fast Advanced Master Mix (Life Technologies) and analyzed on the QuantStudio 7 Flex Real-Time PCR system (Life Technologies). PCR primers used in this study were included in the Assay on-Demand Gene Expression Assay Mix (Life Technologies). The following program settings were used for amplification: 50 °C for 2 min, 95 °C for 2 min, then 40 cycles of 95 °C for 1 s and 60 °C for 20 s. The quantitative values were obtained from the average threshold cycle (Ct) number of each sample run in triplicate and gene expression analysis performed using the comparative Ct method. Target gene expression was normalized against RPS18 mRNA expression for each sample and expressed relative to the indicated control.

#### *Ex vivo* and *in vitro* cell culture treatments

Splenocytes were extracted from WT and TLR7 KO spleens by mashing the tissue through a 40 μm mesh filter and washing with sterile RPMI-1640 media (Thermofisher Scientific). Red blood cells were then lysed in ACK lysis buffer and splenocytes cultured in complete RPMI-1640 media supplemented with Glutamax, fetal bovine serum (FBS; 10%) and penicillin-streptomycin (1%) for treatments. T cells from WT and TLR7 KO splenocyte suspensions were isolated using the mouse Pan T cell Isolation Kit II (Miltenyi Biotec). Splenocytes (1x10^6^) or T cells (3x10^5^) were seeded per well of a U-bottom 96-well plate in 200 μL complete media containing 10 μg/mL IMQ (Invivogen), PR8 virus (MOI 1), 1 μM phorbol 12-myristate 13-acetate (PMA) plus 0.5 μM ionomycin, or 0.1 ng/mL recombinant murine IFNγ (Peprotech), and incubated for 24 h. T cell culture media also contained 15 U/mL hIL-2 (Sigma-Aldrich). Cells were then resuspended in FACS buffer and stained with fluorescently conjugated antibodies as described earlier.

PD-L1 expression was also measured in immortalized bone marrow-derived macrophages (BMDMs) and alveolar MH-S macrophages. BMDMs were maintained in complete DMEM supplemented with L-glutamine, glucose (4500 mg/L), sodium pyruvate (110 mg/L), and FBS (10%) while MH-S cells were cultured in complete RPMI-1640 media as described above. For treatments, 8x10^4^ cells were seeded in 24-well plates the day before and then exposed to 10 μg/mL IMQ, PR8 virus (MOI 1) or 0.1 ng/mL recombinant murine IFNγ for 24 h. In some experiments, cells were incubated in BALF for 1 h before being replaced with complete media for the remaining 23 h. Cells were then surface-stained and processed for flow cytometry as described earlier.

For the treatment of lung cell suspensions, 1x10^5^ cells from digested WT or TLR7 KO lung tissues were seeded in each well of a 96-well plate containing complete DMEM. Cells were then exposed to 10 μg/mL IMQ, PR8 virus (MOI 1) or left untreated for 18 h. Following treatment, media was aspirated and cells directly lysed for RNA extraction and subsequent gene expression analysis.

#### Cytokine protein levels by ELISA

Enzyme-linked immunosorbent assays (ELISA) were performed on bronchioalveolar lavage fluid (BALF) or cell culture supernatant using the mouse IFN-gamma or IL6 DuoSet ELISA kits (R&D System, Minneapolis, MN). To isolate BALF, a small incision on trachea was made and a sheathed 21-Gauge needle was inserted into the lumen. The lung was then lavaged with 300–400 μL aliquots of PBS repeatedly with gentle massaging of the chest with each aspirate transferred collected until a volume of 1 mL was collected. Enumerated cells were pelleted and cleared fluid stored at −80 °C until use. One hundred microliters of BALF or culture supernatant was added in duplicate to pre-coated 96-well plate and incubations performed according to manufacturer’s instructions. The 96-well plate was read on the CLARIOstar (BMG) at a wavelength of 450 nm. Cytokine titers in the samples were determined by plotting the optical densities, using a four-parameter fit for the standard curve and expressed in pg/mL.

### Quantification and statistical analysis

All data are expressed as the mean ± SEM. Statistical analyses were performed using GraphPad Prism (GraphPad Software Version 8.2, San Diego CA, USA) using two-way ANOVA with Tukey’s post-hoc tests for multiple comparisons. A *p*-value of less than 0.05 was considered statistically significant. Statistical details of experiments including n numbers can be found in figure legends.
