# Supplementary material for: TLR7-mediated inflammation drives PD-L1 upregulation and T cell exhaustion during influenza A virus infection
Source: iScience. 2026 Jan 22;29(2):114776. doi: 10.1016/j.isci.2026.114776 (PMC12907126; doi:10.1016/j.isci.2026.114776)
Supplement: Document S1. Figures S1–S5 [file mmc1.pdf]

iScience, Volume *29*

Supplemental information

TLR7-mediated inflammation drives PD-L1 upregulation and T cell exhaustion during influenza A virus infection

Mark A. Miles, Stella Liong, Felicia Liong, John J. O'Leary, Doug A. Brooks, and Stavros Selemidis

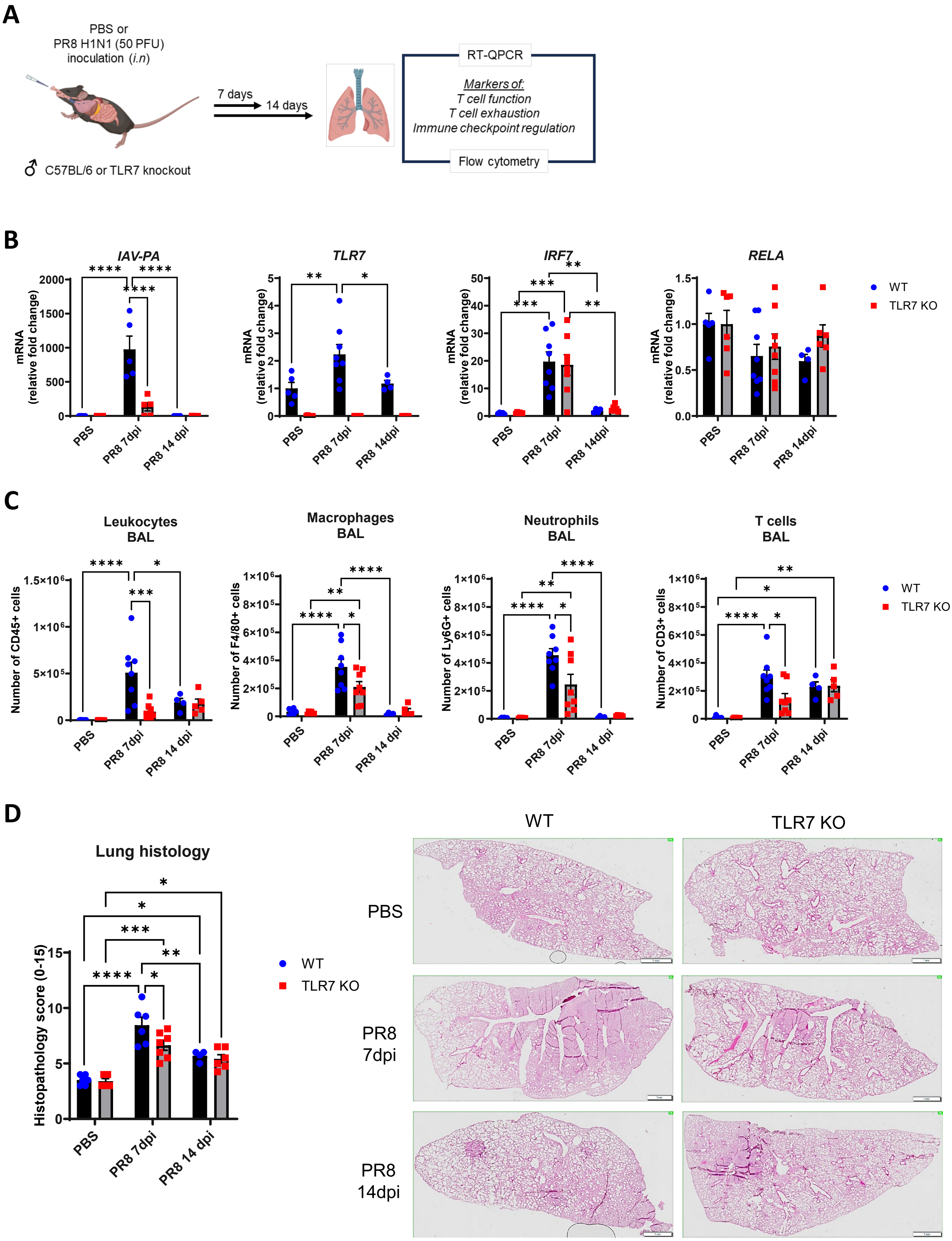

Figure S1. TLR7 KO mice exhibit reduced viral load, airway inflammation and lung pathology following IAV infection. WT C57BL/6 or TLR7 KO mice were intranasally infected with PR8 (50 PFUs) or mock infected with PBS, and lung tissue harvested after 7 or 14 days. (A) Schematic representation of the animal experimentation protocol and lung tissue analysis performed and presented in the main figures is shown. (B) Lung mRNA levels of viral *PA, TLR7, IRF7* and *RELA* were measured by RT-qPCR and expressed relative to RPS18 housekeeping as a fold-change above uninfected controls of each mouse genotype. (C) Immune cells infiltrating the airways were collected by bronchioalveolar lavage (BAL) and absolute number of leukocytes (CD45+), macrophages (CD45+ F4/80+), neutrophils (CD45+ Ly6G+) or T cells (CD45+ CD3+) was determined by flow cytometry relative to live cell counts. (D) Lung histopathological analysis for each individual mouse was assessed following H&E staining of 4 μm longitudinal sections of paraffin embedded lung tissue. The total histopathology score was calculated by combining a 0-5 severity score for the following three parameters: alveolitis (alveolar inflammation of parenchyma), total inflammatory cell infiltrate, and peribronchiolar inflammation (inflammation around bronchiolar airway wall). Representative images are shown, with a scale bar of 1 mm indicated. Data are expressed as mean ± SEM, n = 4-8 mice per experimental group. Statistical analysis was conducted using two-way ANOVA test followed by Tukey's post hoc test for multiple comparison test (*p < 0.05, **p < 0.01, ***p < 0.001, ****p < 0.0001).

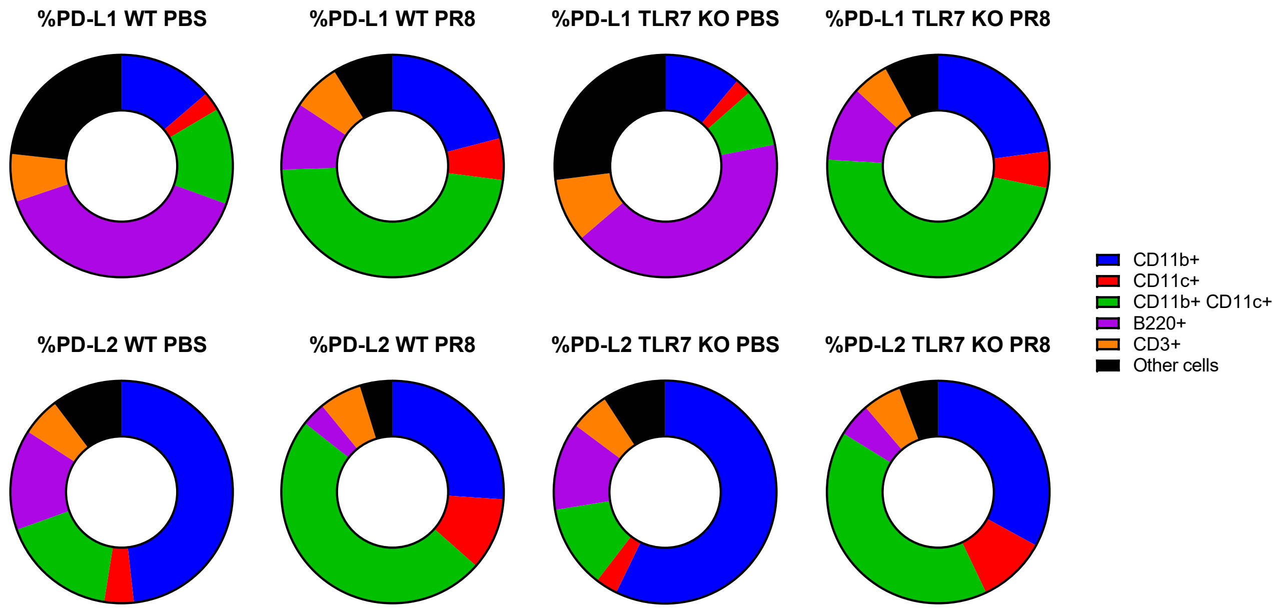

Figure S2. Composition of lung immune cells expressing PD-L1 or PD-L2 following IAV infection is unaffected by TLR7 deletion. WT C57BL/6 or TLR7 KO mice were intranasally infected with PR8 (50 PFUs) or mock infected with PBS. After 7 days, the frequencies of immune cells (CD45+) expressing PD-L1 or PD-L2 in the lungs were measured by flow cytometry. Data represent the average of 6-8 mice per experimental group.

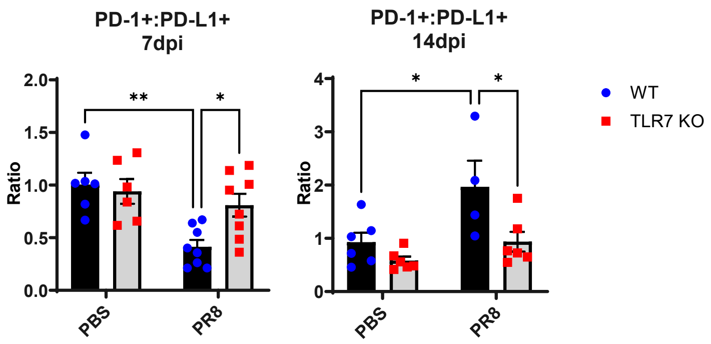

Figure S3. TLR7 deficiency alters the PD-1:PD-L1 ratio in the lungs. Ratios of PD-1 and PD-L1 surface expression in cells of the lungs from PBS (mock) or PR8 infected (50 PFUs) WT C57BL/6 or TLR7 KO mice. Data are expressed as mean ± SEM, n = 6-8 mice per experimental group. Statistical analysis was conducted using two-way ANOVA test followed by Tukey's post hoc test for multiple comparison test (*$p < 0.05$, **$p < 0.01$).

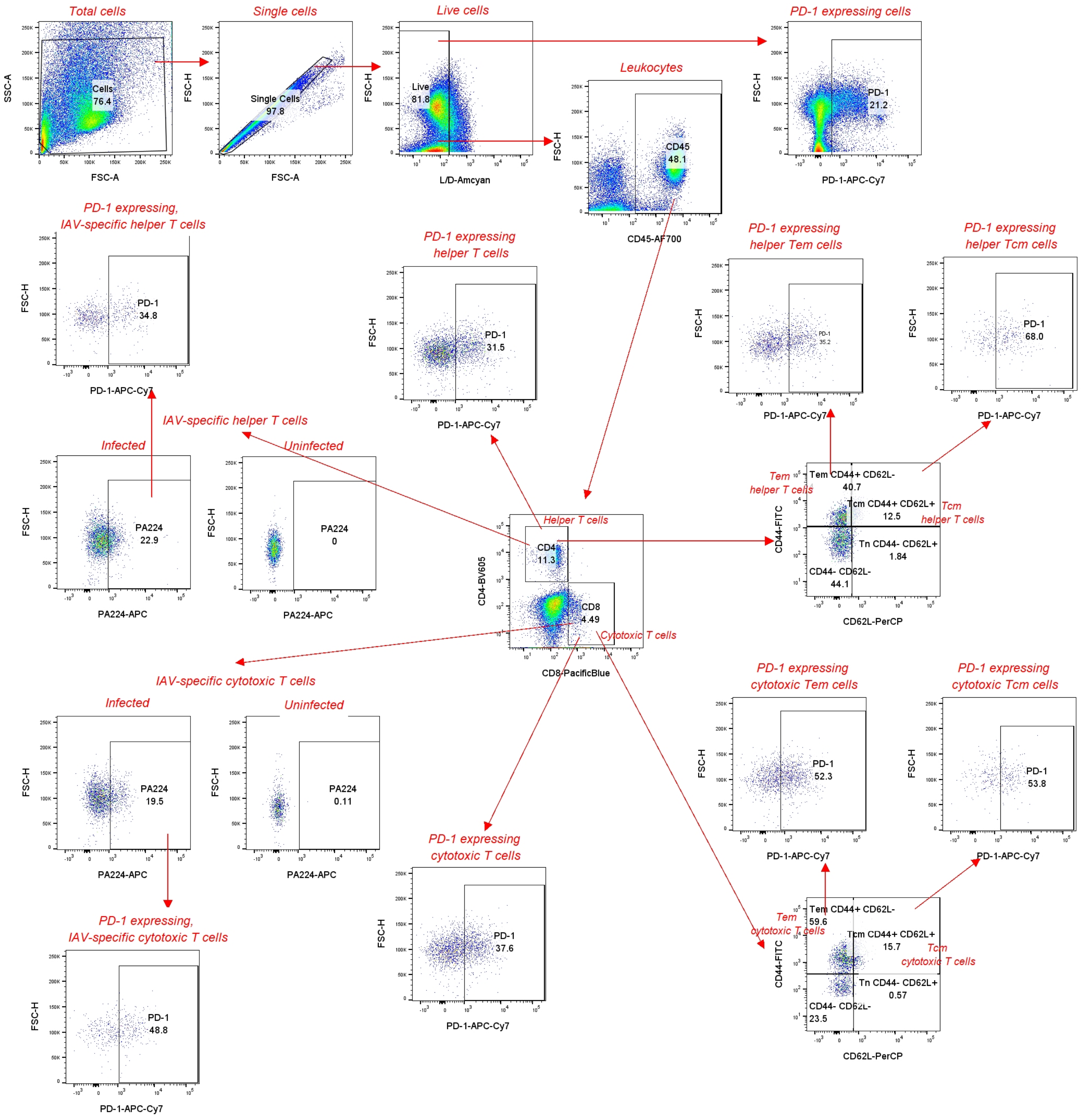

Figure S4. Flow cytometry gating strategy for PD-1 expression.

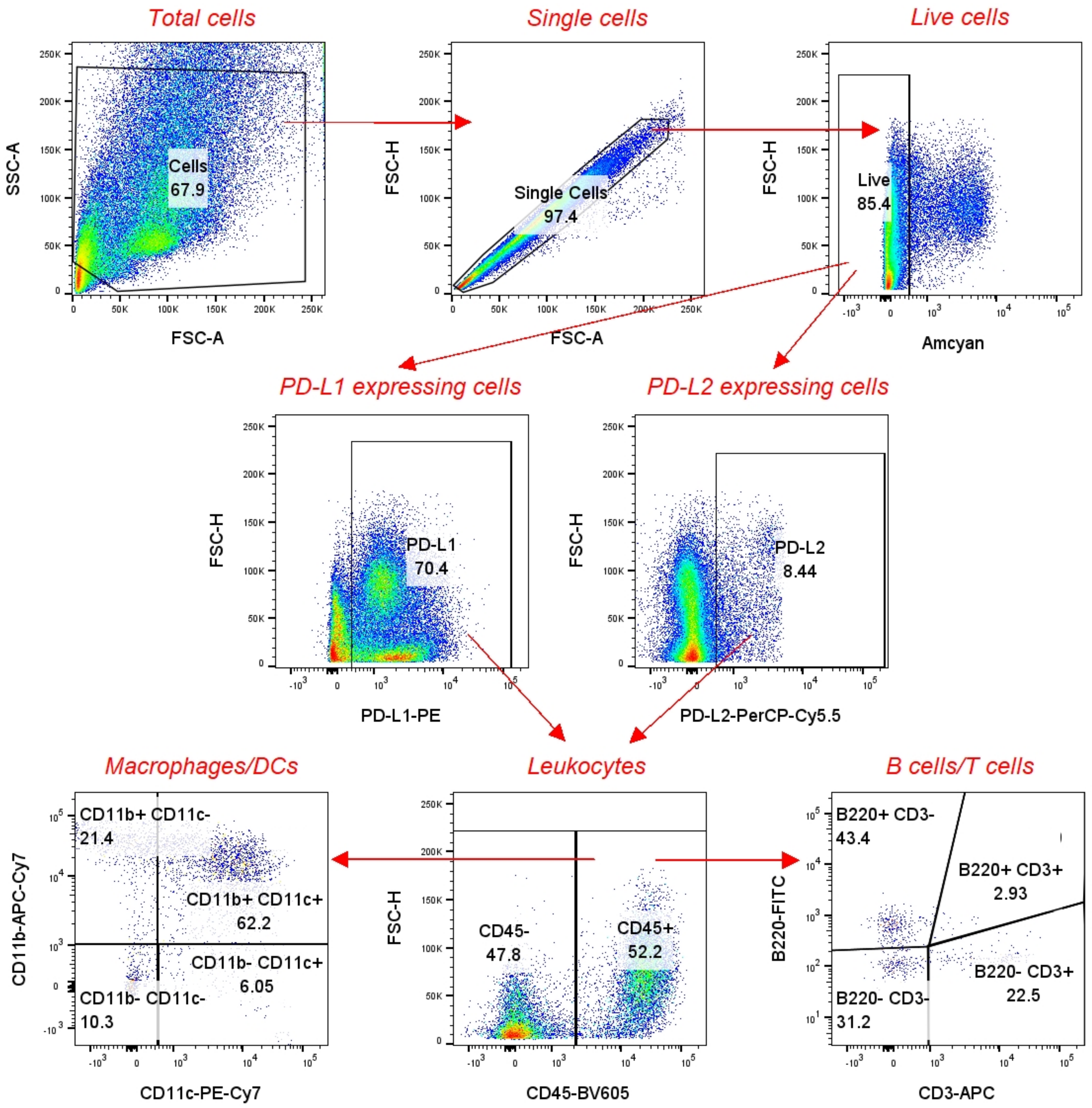

Figure S5. Flow cytometry gating strategy for PD-L expression.
